# Membrane estrogen receptor-α levels predict estrogen-induced ERK1/2 activation in MCF-7 cells

**DOI:** 10.1186/bcr959

**Published:** 2004-11-26

**Authors:** Dragoslava Zivadinovic, Cheryl S Watson

**Affiliations:** 1 Department of Human Biological Chemistry and Genetics, University of Texas Medical Branch, Galveston, Texas, USA

**Keywords:** membrane estrogen receptor-α, MCF-7 human breast cancer cells, extracellular regulated protein kinase

## Abstract

**Introduction:**

We examined the participation of a membrane form of estrogen receptor (mER)-α in the activation of mitogen-activated protein kinases (extracellular signal-regulated kinase [ERK]1 and ERK2) related to cell growth responses in MCF-7 cells.

**Methods:**

We immunopanned and subsequently separated MCF-7 cells (using fluorescence-activated cell sorting) into mER-α-enriched (mER^high^) and mER-α-depleted (mER^low^) populations. We then measured the expression levels of mER-α on the surface of these separated cell populations by immunocytochemical analysis and by a quantitative 96-well plate immunoassay that distinguished between mER-α and intracellular ER-α. Western analysis was used to determine colocalized estrogen receptor (ER)-α and caveolins in membrane subfractions. The levels of activated ERK1 and ERK2 were determined using a fixed cell-based enzyme-linked immunosorbent assay developed in our laboratory.

**Results:**

Immunocytochemical studies revealed punctate ER-α antibody staining of the surface of nonpermeabilized mER^high ^cells, whereas the majority of mER^low ^cells exhibited little or no staining. Western analysis demonstrated that mER^high ^cells expressed caveolin-1 and caveolin-2, and that ER-α was contained in the same gradient-separated membrane fractions. The quantitative immunoassay for ER-α detected a significant difference in mER-α levels between mER^high ^and mER^low ^cells when cells were grown at a sufficiently low cell density, but equivalent levels of total ER-α (membrane plus intracellular receptors). These two separated cell subpopulations also exhibited different kinetics of ERK1/2 activation with 1 pmol/l 17β-estradiol (E_2_), as well as different patterns of E_2 _dose-dependent responsiveness. The maximal kinase activation was achieved after 10 min versus 6 min in mER^high ^versus mER^low ^cells, respectively. After a decline in the level of phosphorylated ERKs, a reactivation was seen at 60 min in mER^high ^cells but not in mER^low ^cells. Both 1A and 2B protein phosphatases participated in dephosphorylation of ERKs, as demonstrated by efficient reversal of ERK1/2 inactivation with okadaic acid and cyclosporin A.

**Conclusion:**

Our results suggest that the levels of mER-α play a role in the temporal coordination of phosphorylation/dephosphorylation events for the ERKs in breast cancer cells, and that these signaling differences can be correlated to previously demonstrated differences in E_2_-induced cell proliferation outcomes in these cell types.

## Introduction

Estrogen receptor (ER)-α has traditionally been defined as a ligand-dependent transcription factor that regulates its target genes by binding to estrogen response elements present in the promoters of many responsive genes [1]. However, an ever-increasing number of reports indicate that the cellular actions of estrogens can be initiated at the plasma membrane, through membrane versions of estrogen receptors (mERs) [2-4] or via other membrane-resident 17β-estradiol (E_2_)-binding proteins [5]. There is also evidence that mER-α from vascular endothelium and human MCF-7 breast cancer cells is localized in specialized cholesterol-rich membrane microstructures (caveolae), where it can associate with different signaling molecules and participate in various nongenomic actions [6,7].

A variety of rapid E_2_-induced signal transduction events can lead to stimulation of calcium flux, cAMP production, phospholipase C activation, and inositol phosphate production [8]. Mitogen-activated protein kinases (MAPKs) such as extracellular signal-regulated kinase (ERK)1 and ERK2 are also rapidly stimulated by estrogens in various cell types (e.g. endothelial [9], osteoblastic [10], neuroblastoma [11], and breast cancer cells [12]). However, the specific relationship of these responses to the levels of antibody-identified ER-α in the membrane has rarely been investigated [13,14]; other rapid estrogen-induced actions were specifically linked to mER-α in pituitary tumor cells in our previous studies [15-18].

The two isoforms of ERK (p42 and p44) play critical roles in the control of cell proliferation, differentiation, homeostasis, and survival. Traditionally, autophosphorylation of receptor tyrosine kinases after ligand binding initiates the cascade of phosphorylation steps that result in dual ERK phosphorylation (on Thr202 and Tyr204 in the human enzyme, or Thr183 and Tyr185 in the rat enzyme [19]). The signaling pathway initiated by E_2 _at the level of the plasma membrane is not yet completely understood, although recent studies have implicated a cascade of intermediary proteins and signaling steps involving mER-α, G-proteins, Src-induced matrix metalloproteinases that liberate heparin-binding epidermal growth factor (EGF), and EGF receptor [13]; the involvement of many other signaling pathways remains unexamined.

Whether different levels of mER can influence signaling parameters (such as kinetics and final levels of second messengers) that lead to physiological responses remains to be investigated. To address this question we separated MCF-7 cells into two subpopulations based on outer membrane-exposed mER-α levels and confirmed their differential mER-α expression by several methods. We investigated the association of mER-α with caveolin-rich membrane fractions in cells enriched for membrane display of these receptors. We then linked the level of mER-α to the magnitude and patterns of E_2_-induced ERK1/2 activation. Although activation of kinases was previously demonstrated, those other studies did not address the accompanying inactivation mechanisms for ERKs involving several specific cellular phosphatases.

## Methods

### Cell immunoseparation and subculturing

Our MCF-7 cells originated from the Michigan Cancer Center. We separated them into two subpopulations by immunopanning [16,20] using C-542 carboxyl-terminal ER-α antibody provided by Drs Dean Edwards and Nancy Weigel; this antibody is now commercially available from Stressgen Biotechnologies (Victoria, Canada). Briefly, sterile antibody on the surface of a petri plate bound cells over a 1-hour time period at 4°C, and cells that attached to the plate (mER^+^) were propagated separately from those that did not bind (mER^low^). The mER^+ ^cells were then subjected to further selection via fluorescence-activated cell sorting (FACS) using the same antibody [21]. Cells undergoing sequential separation were highly enriched for mER-α (mER^high^). All cell subpopulations were routinely cultured in phenol-red free Dulbecco's modified eagle medium (Gibco-BRL, Grand Island, NY, USA) supplemented with 10% heat-inactivated DSCS (defined/supplemented bovine calf serum; HyClone Laboratories Inc., Logan, UT, USA) and 1% of an antibiotic–antimycotic (Gibco Invitrogen Corporation, #15240-062). Cells used in the experiments were between passage 8 and 14 after separation.

Three days before each experiment, the cell growth medium was replaced with medium containing 4 × dextran-coated charcoal stripped serum (DCSS medium) or completely defined medium (DM; phenol-red free Dulbecco's modified eagle medium with 5 μg/ml each of insulin and transferrin, 5 ng/ml selenium, and 0.1% bovine serum albumin). Stripped serum was produced by incubation with an equivalent volume of packed 0.25% weight/vol charcoal Norit A for 2 hours on a shaker at 4°C. The charcoal preparation had previously been suspended in 0.0025% (weight/vol) dextran T-70 (Sigma-Aldrich, St. Louis, MO, USA) in 0.25 mol/l sucrose, 1.5 mmol/l MgCl_2_, and 10 mmol/l HEPES (pH 7.6). The charcoal was pelleted at 500 *g *for 10 min, the supernatant (stripped serum) decanted, and the process repeated a total of four times.

### Fluorescence immunocytochemical detection of membrane ER-α

The protocol published for immunocytohcemical detection of mER-α in the GH_3 _rat pituitary cell line [22] was optimized for MCF-7 cells. Briefly, the cells were fixed in 2% paraformaldehyde/0.1% glutaraldehyde in phosphate-buffered saline for 25 min at room temperature; those conditions preserve the integrity of the cell membrane and enable detection of the specific ER-α antibody bound to the cell surface without interference from the usually more intense nuclear signal. Autofluorescence was reduced by blocking the aldehyde groups for 15 min with 1% Na_2_HPO_4 _and 0.05% NaBH_4_. Nonspecific binding sites were blocked with 0.1% fish gelatin in phosphate-buffered saline for 45 min at room temperature, which we successfully used previously in GH6/B6/F10 cells [23,24]. For the present studies with MCF-7 cells, we compared the nonspecific antibody signal blocking abilities of fish gelatin, horse serum, bovine serum albumin and nonfat dry milk, all commonly used methods, and found them to be equally useful.

The plates were incubated with C-542 antibody (5–10 μg/ml) overnight at 4°C in the blocking solution. To detect the bound primary antibody and amplify the signal, we used a biotinylated secondary antibody from the biotin/avidin Vectastain ABC-Alkaline Phosphatase kit and Vector red as a substrate, together with 40 μl of 125 mmol/l levamisol (which inhibits the endogenous alkaline phosphatases). All the components for detection of bound primary antibody were obtained from Vector Laboratories Inc. (Burlingame, CA, USA). Fluorescence photography was performed as previously described [15,22]. The images were photographed with Kodak HC4000 color film and a camera (Model C-35AD-4) with Olympus AHBT microscope and fluorescence attachment (model AH2-RFL) using the FITC filter, under 100 × magnification. Digital deconvolution and pseudo-coloring were performed with Image-Pro Plus software applied to images captured through the FITC filter with a CoolSNAP-Pro digital monochrome camera and a ProScan motorized stage (Meyer Instruments, Houston, TX, USA) attached to a Olympus AHBT microscope equipped with fluorescence attachments (model AH2-RFL).

### Quantification of membrane ER-α

The mER was quantified with a protocol modified from one previously developed in our laboratory for GH_3 _cells [23]. Briefly, cells plated and treated in 96-well plates were fixed as described for immunocytochemistry (see above), and the integrity of the membrane was verified by lack of staining with the anti-clathrin antibody (ICN Biomedicals, Aurora, OH, USA), as clathrin is localized just inside the plasma membrane. Different concentrations of C-542 ER-α antibody were tested (in the range 1–12 μg/ml), and the tagging enzymatic reaction (alkaline phosphatase) was monitored for different time intervals (5, 15 and 30 min) in order to determine optimal conditions for measurement. The specificity of the C-542 antibody was checked by comparing its binding with the nonspecific binding of mouse IgG_1k _(mIgG_1k_, of the same immunoglobulin isotype) and by the ability of the peptide representing the C-542 epitope (Genosys, Woodlands, TX, USA) to decrease C-542 binding. Other controls included incubation without any antibody to detect endogenous phosphatase not inhibited by levamisol, and without primary antibody to detect nonspecific binding of secondary antibody. The total cellular ER-α was measured by applying the same procedure to cells permeabilized by including 0.1% of the non-ionic detergent IGEPAL CA-630 (Sigma-Aldrich) during the fixation procedure. The absorbance of the alkaline phosphatase product paranitrophenol (pNp) in each well was measured at 405 nm and normalized against the number of cells determined by the absorbance of crystal violet (CV) at 590 nm, as previously described [23].

### Caveolae preparation and Western analysis

To concentrate caveolin-rich membranes, we extended a previously published protocol [25] by introducing a dialysis step to remove sucrose, and a vacuum spin at a low drying rate to concentrate the samples. Specifically, cells were seeded in three 150 mm diameter plates and grown in serum-supplemented medium until 60% confluent. The growth medium was replaced with DCSS medium devoid of antimycotic compound and cultured for an additional 3 days. Cells from all three plates were collected in 1 ml lysis buffer consisting of 50 mmol/l Tris/HCl (pH 7.5), 5 mmol/l EDTA, 100 nmol/l NaCl, 50 mmol/l NaF, 1 mmol/l PMSF, 0.2% TritonX-100 and protease inhibitor cocktail P8340 (100 × diluted; Sigma-Aldrich). Cells in solution were passed through a 25-g syringe needle, then homogenized with 25 strokes using a Dounce B-type pestle. The homogenate was adjusted to 45% sucrose by addition of an equivalent volume of 90% sucrose. A discontinuous sucrose gradient consisting of the sample, 35% sucrose, and a top layer of 5% sucrose was centrifuged for 18 hours at 200,000 *g*. Fractions of 1 ml were collected and checked for the presence of caveolin-1 and caveolin-2 by Western analysis using antibodies from Biosciences (San Jose, CA, USA). Fractions 5 and 6 (the interface between 5% and 35% sucrose) contained the highest amount of caveolin-1. Those two fractions were pooled and dialyzed overnight against the lysis buffer. The sample was then concentrated by vacuum spin, and 20 μg of these proteins separated by 4–20% SDS-PAGE. The proteins were transferred to nitrocellulose membranes and the mER-α bands were probed with C-542 ER-α antibody. After incubation with secondary antibody (conjugated with horse radish peroxidase) the bands were visualized with an ECL kit (Amersham Pharmacia Biotech, Piscataway, NJ, USA).

### Fixed cell-based enzyme-linked immunosorbent assay detection of activated ERK1/2 in 96-well plates

A protocol previously developed in our laboratory for other cells [26] was optimized for MCF-7 cells. Cells were plated at a density of 4000/well in 96-well plates, and after 24 hours the growth medium was replaced with DCSS medium. After 3 more days of culture the cells were treated with E_2 _(1 pmol/l) for different time intervals (3, 6, 10, 20, 30 and 60 min), or with different E_2 _concentrations (from 10^-16 ^to 10^-7 ^mol/l) for 10 and 6 min for mER^high ^and mER^low ^cells, respectively. After treatments the cells were fixed in 2% paraphormaldehyde/0.21% picric acid for 2 days at 4°C. The cells were subjected to a 60 min blocking step with 0.1% fish gelatin and 0.1% Triton X-100 at room temperature. Incubation with the antibody raised against the phosphorylated forms of ERK1/2 (Cell Signaling Technology, Beverly, MA, USA) was performed overnight at 4°C (1:400 dilution). To quantify active ERKs, biotinylated secondary antibody (anti-mouse/anti-rabbit) conjugated to alkaline phosphatase was applied. Substrate pNp phosphate was added and incubated for 25 min at 37°C, and the absorbance of the pNp product was determined at 405 nm in a plate reader (Wallac 1420; Perkin Elmer, Boston, MA, USA). The levels of phosphorylate (pERK1/2) were normalized to the cell number in each well (measured using the CV assay).

To confirm the activation of ERK1/2 (after 3, 6 and 10 min in mER^high ^or 6 min in mER^low^), we pretreated the cells for 15 min with 40 μmol/l U0126 MEK1/2 inhibitor (Cell Signaling Technology). The ER antagonist ICI182,780 (Tocris Cookson Inc., Ellisville, MO, USA) at a concentration of 1 μmol/l was tested with or without E_2 _by preincubating the cells with ICI182,780 for 30 min followed by the addition of 1 pmol/l E_2 _or by simultaneous addition of ICI182,780 and E_2_. MDA-MB-231 cells used to test the requirement of ER-α for these responses were obtained from ATCC (Manassas, VA, USA). We confirmed the absence of ER-α mRNA in these cells by multiple reverse transcription polymerase chain reaction primer pairs representing the ER-α sequence (data not shown).

### Cell proliferation

Cells were plated at a density of 1000 cells/well in 96-well plates. The next day the growth medium was replaced with DCSS containing different treatments. The 1 pmol/l E_2 _was present either for the duration of the experiment or as a short pulse treatment (10 min), whereas 1 pmol/l E_2_-peroxidase (Sigma-Aldrich; concentration based on the E_2 _content of the conjugate) was used only as short pulse treatment. The effect of MEK inhibitor (40 μmol/l U0126) on the pulsed E_2_-induced proliferation of mER^high ^cells was tested by a pretreatment for 15 min and an additional treatment for 10 min with E_2 _together with the inhibitor. To block the effect of E_2 _in the pulse treatment, we used ER-α antibody (AER315; NeoMarkers Inc., Fremont, CA, USA) raised against the ligand-binding domain. The cells were pretreated with 1 μg/ml AER315 antibody for 1 hour at room temperature, followed by a 10 min incubation at 37°C with 1 pmol/l E_2 _in the presence of the antibody. Controls for E_2 _treatment were done on a separate plate, because we previously determined that low amounts of volatilized estrogens can affect responses mediated via nongenomic signaling pathways [24]. After 5 days the cells were fixed with 2% paraformaldehyde/0.1% glutaraldehyde in phosphate-buffered saline in preparation for the CV assay (see below).

### Crystal violet assay

The number of the cells in each well was determined with the CV assay [27], which we modified previously [23]. Fixed cells were incubated in 0.1% filtered CV solution for 30 min at room temperature, and excess dye was removed by three brief rinses with ddH_2_O. The plates were then air dried, the dye was extracted with 10% acetic acid, and the extract (in the same wells) was then read in a plate reader (Wallac 1420, Perkin Elmer) at 590 nm. The utility of this assay was previously verified for GH3B6 cells by comparison with other assays of cell number in combination with the immunoplate assays [23]. In addition, for MCF-7 cells we verified the utility of this assay for measuring cell number by comparison with DNA content measurements and with cell counts by hemocytometer (data not shown). We also compared the CV assay with the MTT assay, which is often used to determine viable cell number (ATCC), and we obtained a linear correlation for the two assays. These latter results are presented in the accompanying paper [28].

### Statistical analysis

Statistical differences between two sets of data were determined using two-way analysis of variance. The differences between the entire curves were tested by comparing the sum of squares of the residuals from each individual curve with the sum of squares of the residuals of the combined curve by applying a Microsoft Excel F test. *P *< 0.05 was considered statistically significant.

## Results

### Immunoseparated cell characterization of mER-α

**Figure 1:**
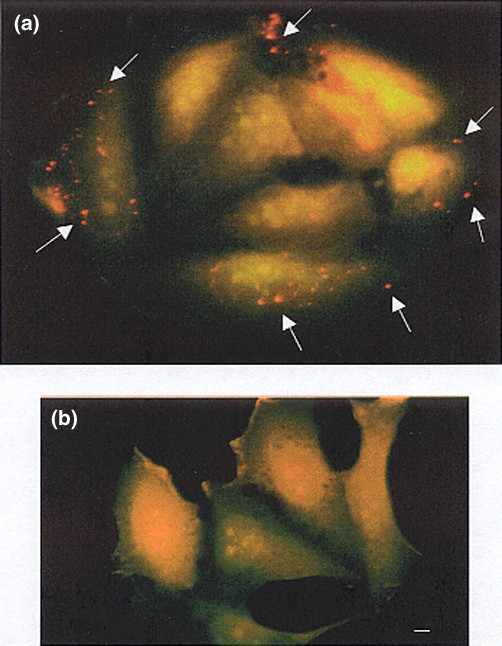
Fluorescence immunocytochemical detection of membrane estrogen receptor (mER)-α in nonpermeabilized MCF-7 cells. Cells were fixed with 2% pararaformaldehyde/0.1% glutaraldehyde, probed with C-542 carboxyl-terminal estrogen receptor-α antibody, and visualized with a biotinylated secondary antibody–avidin conjugated alkaline phosphatase fluorescent Vector red product. Fluorescence images were photographed using the FITC filter and 100 × magnification. **(a) **mER-α-enriched (mER^high^) MCF-7 cells; the arrows indicate some of the punctate staining. **(b) **mER-α-depleted (mER^low^) MCF-7 cells have no staining. The bar in panel b represents 10 μm.

**Figure 2:**
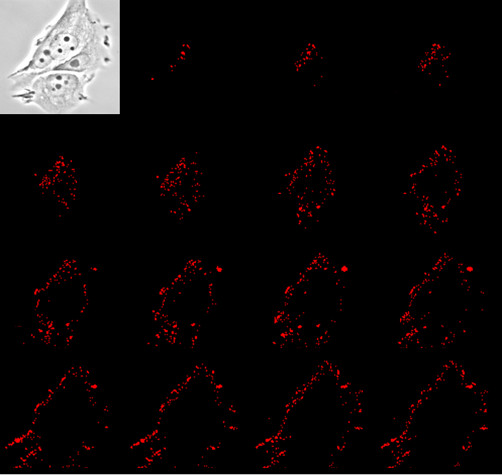
Digital deconvolution of membrane estrogen receptor (mER)-α fluorescent image in mER-α-enriched (mER^high^) MCF-7 cells. Fifteen different focal planes starting from the top of the cells and moving toward the bottom are displayed. The sequential slices are from left to right in each row of images. These cells are nonpermeabilized and treated as described in the previous figure legend. The corresponding transmitted light image is displayed in the upper left-hand corner. FITC filter images were captured, pseudo-colored, and processed by digital deconvolution.

Immunopanning and subsequent FACS successfully separated MCF-7 cells into two populations according to the expression of mER-α observed in immunocytochemistry experiments. Punctate staining can be seen on the surface of unpermeabilized mER^high ^cells (Fig. 1a), whereas the majority of mER^low ^cells did not exhibit this staining (Fig. 1b). Whenever occasional staining was present on cells in the mER^low ^population, its appearance was similar to that seen on mER^high ^cells (data not shown). Secondary antibody staining alone was at levels similar to that shown for the mER^low ^cells in Fig. 1b (not shown). When permeabilized, both subpopulations of cells exhibited plentiful cytoplasmic and nuclear staining (not shown) at similar levels. Digital deconvolution (in 15 separate cell planes) performed on a grouping of three unpermeabilized mER^high ^cells clearly demonstrated punctuate staining all along the periphery of these cells (Fig. 2).

**Figure 3:**
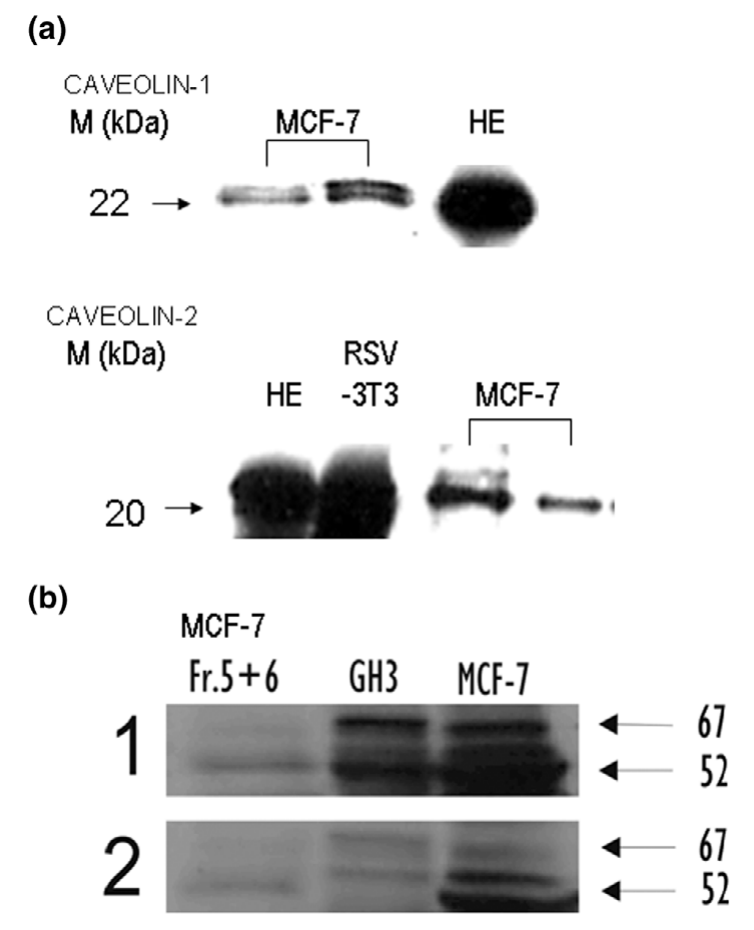
Identification of membrane estrogen receptor (mER) in caveolae by Western blot analysis. **(a) **Caveolin-1 (upper panel) and caveolin-2 (lower panel) in MCF-7 cell membranes. Membrane fractions containing caveolae were separated on discontinuous sucrose gradients. Fractions 5 and 6 were combined and 20 and 40 μg/ml protein (shown by adjacent lanes with connecting bar) were resolved on a 4–20% SDS-polyacrylamide gel, transferred to a nitrocellulose membrane, and probed with specific antibodies. Fractions from human endothelial (HE) and mouse fibroblast (RSV-3T3) cells were used as positive controls (50 μg/ml protein). **(b) **Whole cell lysates (GH_3_, MCF-7) and pooled membrane fractions 5 and 6 (Fr.5+6) from sucrose gradients for MCF-7 cells were probed with estrogen receptor-α-specific antibody C-542 (panel 1) or the same antibody blocked with the epitope peptide (panel 2). The arrows show the positions of major bands that were identified as receptor by epitope competition (at 67 and 52 kDa).

To determine whether mER-α is in a submembrane location in our mER-α-enriched cells, we colocalized mER-α with caveolin proteins in gradient-separated membrane fractions. Our mER^high ^cells express both caveolin-1 (form α and β represented by doublet bands) and caveolin-2 (Fig. 3a; two different concentrations of protein were loaded in adjacent lanes). In the same density gradient fractions that contained the majority of these caveolar structural proteins (fractions 5 and 6), mER-α was found (Fig. 3b). We detected several prominent bands from both the sucrose gradient fractions (fractions 5 and 6) and the whole cell lysates (Fig. 3b, panel 1). To determine those bands whose signal was specific for C-542 ER-α antibody, we performed an epitope competition (preblocking of antibody with peptide representing the epitope). We identified two ER-α bands competable with peptide: the 67 kDa (the size of classical ER-α) and a prominent lower band at 52 kDa (Fig. 3b, compare panels 1 and 2).

**Figure 4:**
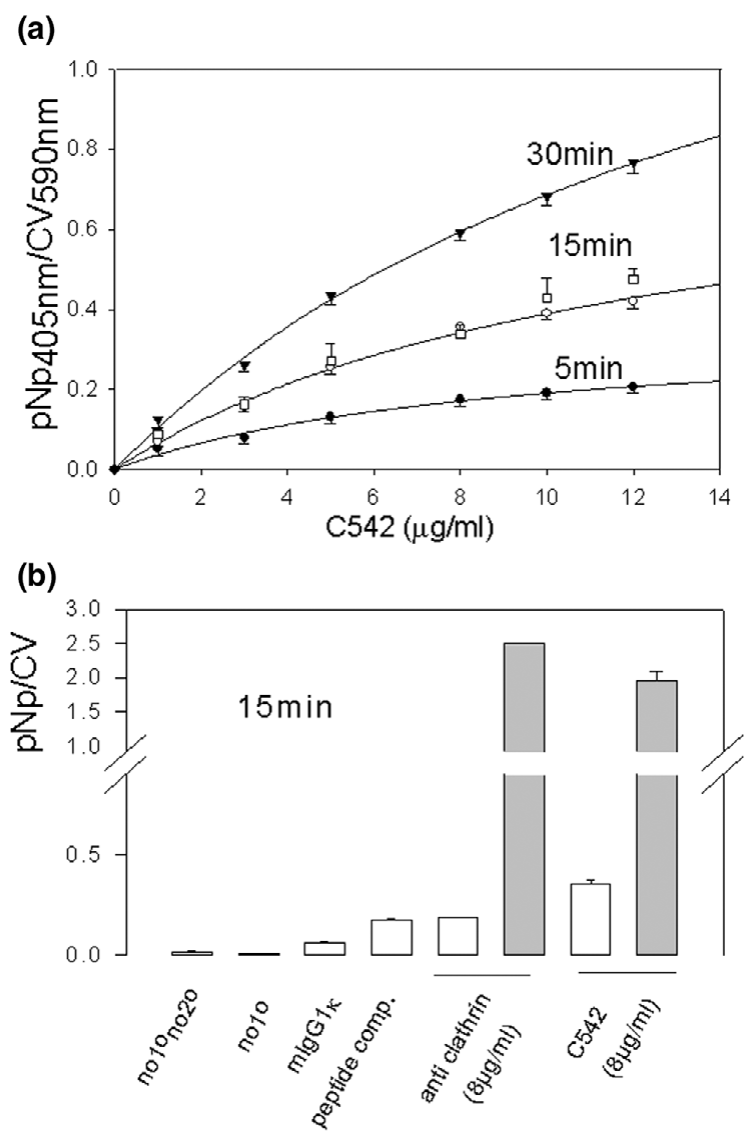
Optimization of 96-well plate immunoassay for membrane estrogen receptor (mER) quantification in MCF-7 cells. **(a) **Different concentrations of estrogen receptor-α-specific C-542 antibody (1–12 μg/ml) were tested. Enzymatic paranitrophenol phosphate (pNpp) hydrolysis yielding the paranitrophenol (pNp) product was monitored at 37°C for 5, 15 and 30 min, as shown. Cells pretreated for 72 hours with medium containing dextran-coated charcoal-stripped serum (DCSS) versus defined medium (DM) are represented with closed and open circles, respectively. **(b) **Antibody binding compared in nonpermeabilized (open bars) and permeabilized (shaded bars) conditions. no1°no2° represents primary and secondary antibodies omitted; no1° represents primary antibody omitted (only secondary antibody applied); mIgG1k represents mouse IgG_1k _isotype control; peptide comp. represents C-542 epitope peptide blocking of the interaction with primary antibody; anti-clathrin represents our test for cell membrane integrity. Values are expressed as means ± standard error. CV, crystal violet.

To quantify mER-α with our plate assay, we first determined the lowest optimal C-542 antibody concentration that would saturate binding to the membrane form of the receptor, and then optimized assay conditions to give a reliably detectable signal in the linear range of the fluorescent product assay. All three signal generation incubation times (5, 15 and 30 min) gave acceptable data, but 15 min assays allowed clear delineation of antigen-saturating concentrations of C-542 antibody utilizing lower antibody concentrations (8 μg/ml; Fig. 4a). Those conditions were used for subsequent assays. The same level of mER-α could be detected in cells kept for 3 days in a completely defined medium lacking serum or in the same medium supplemented with 10% DCSS (Fig. 4a, open circles and closed circles, respectively). The background values (obtained in the absence of primary and secondary antibody, or with no primary antibody) were very low (Fig. 4b). At 8 μg/ml the isotype control mIgG_1k _yielded a value only 10% of that for C-542 antibody; peptide competition resulted in an approximate 50% decrease in C-542 antibody binding, again verifying the specificity of the antibody for this epitope. This verified that the assay was specific, and not subject to interference by endogenous alkaline phosphatases in the presence of levamasole. The fixation conditions that we used preserved the integrity of the cell membrane, as demonstrated by the negligible anti-clathrin antibody binding in nonpermeabilized versus the very high binding in permeabilized cells. Therefore, equivalent protocols differing only in the inclusion of a permeabilization agent during fixation can be used to quantify the total ER (tER) versus the mER.

**Figure 5:**
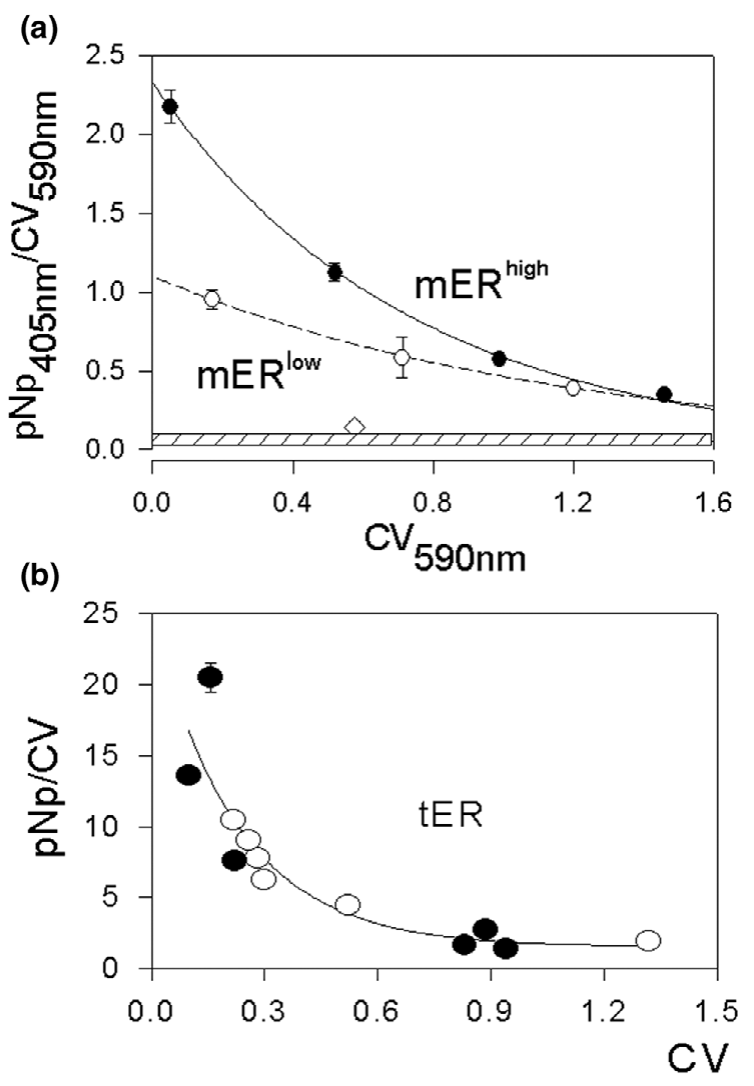
Quantification of membrane estrogen receptor (mER) and total estrogen receptor (tER) in mER-α-enriched (mER^high^) and mER-α-depleted (mER^low^) MCF-7 cells. **(a) **Different numbers of cells were fixed in the same manner as described in Fig. 1 and the level of mER was assessed with 8 μg/ml C-542 antibody. Closed and open circles represent mER^high ^and mER^low ^cells, respectively. The data were approximated with an exponential decay curve and compared by evaluating the difference between the sums of squares of the residuals from each curve with the sum of squares of the residuals for the combined curve using the F-test. The curves are significantly different (*P *= 0.0003). The diamond represents MDA-MB-231 cells and the cross-hatched horizontal bar the level of binding of a mouse IgG_1k _isotype control antibody. **(b) **Level of total ER (intracellular estrogen receptor [iER] plus mER) for mER^high ^(closed circles) and mER^low ^(open circles) under the same binding conditions except for permeabilization of the cells. Where error bars for standard error are not visible, they are smaller than the size of the symbols. CV, crystal violet; pNp, paranitrophenol.

Because we had previously observed an effect of cell density on the level of ER expression in GH_3_/B6/F10 cells, which occurred at a density when cells had just begun to make contact by cell processes [24], we wondered whether the same regulation was applicable to breast cancer cells. Considering this effect, plating a single density may not demonstrate the ability of cells to express mER-α. Therefore, we performed a cell density study for mER^high ^versus mER^low ^breast cancer cell types (Fig. 5). The mER-α signal decreased exponentially with increasing cell number (Fig. 5a). At low plating densities, mER^high ^cells clearly showed much higher levels of mER than did mER^low ^cells (Fig. 5, closed circles and open circles, respectively). The two curves approximating the level of mER were significantly different (*P *= 0.0003). The ER-α-negative cell line MDA-MB-231 did not have mER-α (Fig. 5, diamond), even at relatively low cell density, because their value was at the level of mIgG_1k _isotype control antibody levels (horizontal hatched line). Increased cell density also decreased tER (Fig. 5b); however, mER^high ^and mER^low ^MCF-7 cells exhibited the same level of tER, because all of these data could be approximated with the same curve. Because mER^high ^cells had higher mER-α levels but the same tER-α levels as mER^low ^cells, then, by subtraction, mER^high ^cells have lower intracellular ER-α levels than do mER^low ^cells.

### Kinetics of ERK1/2 activation by 17β-estradiol in MCF-7 cells enriched and MCF-7 cells depleted for membrane ER-α

**Figure 6:**
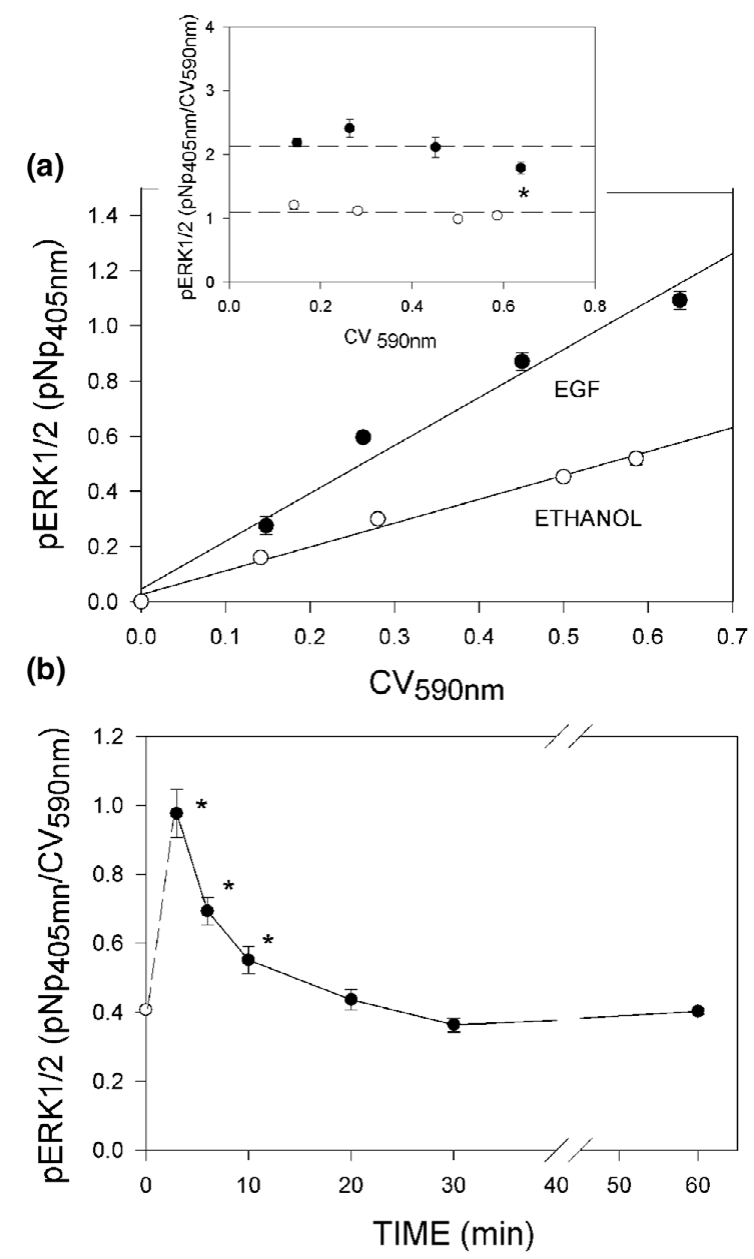
Optimization of fixed cell-based enzyme-linked immunosorbent assay for mitogen-activated protein kinase using epidermal growth factor (EGF) stimulation. **(a) **Different numbers of cells were treated for 10 min with 50 ng/ml EGF, fixed, and the level of extracellular signal-regulated kinase (ERK)1/2 phosphorylation determined using a phospho-specific antibody and the Vectastain ABC alkaline phosphatase kit with paranitrophenol phosphate (pNpp) substrate. Inset: values for paranitrophenol (pNp) normalized against the number of cells determined by the crystal violet (CV) assay. Closed and open circles represent EGF and vehicle treatment, respectively. **(b) **Time course of ERK stimulation by vehicle. Open circle: zero time point estimated by averaging 20, 30 and 60 min values of vehicle treatment. Asterisks indicate significant differences (*P *< 0.05) compared with all other values. Values are expressed as means ± standard error.

During the optimization of the fixed cell-based enzyme-linked immunosorbent assay for ERK activation, we established the optimal cell density for a 10 min EGF treatment, which is known to result in substantial phosphorylation of ERK1/2. Both the controls and EGF-treated cells exhibited increased pERK1/2 with increased cell number (Fig. 6a, main graph). As we expected, normalizing pERK1/2 values to the number of cells (CV absorbance at 590 nm) did not significantly change the ratio values except in the case of the highest number of cells plated, verifying that cells plated in the density range of CV values 0.2–0.6 could be used. Partly because others have shown that ERKs can be activated by mechanical stimulus in MCF-7 cells [29], we tested ethanol-treated controls over the same time course (3–60 min). A pronounced decrease in ERK1/2 phosphorylation was seen with time (Fig. 6b), and so appropriate controls were performed for each time point in all subsequent experiments.

**Figure 7:**
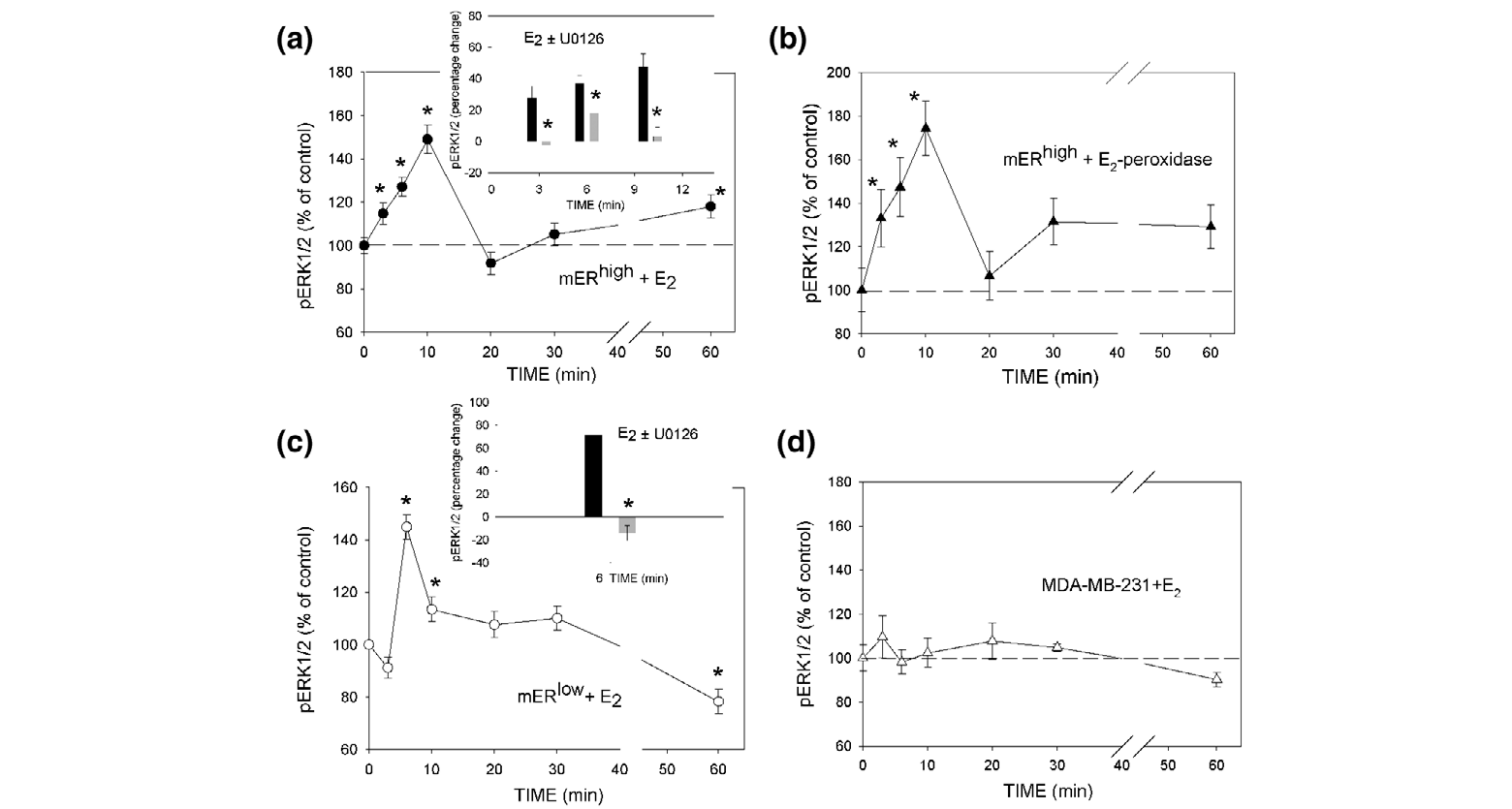
Time course of extracellular signal-regulated kinase (ERK)1/2 activation with 17β-estradiol (E_2_) and impeded ligand in membrane estrogen receptor (mER)-α-enriched (mER^high^) and mER-α-depleted (mER^low^) MCF-7 cells, and receptor-less cells. After 72 hours in medium with dextran-coated charcoal-stripped serum (DCSS), cells were treated with 1 pmol/l E_2_, or an equivalent concentration of hormone contained in an E_2_-peroxidase conjugate, for different time intervals. **(a, b) **mER^high ^MCF-7 cells treated with E_2 _and E_2_-peroxidase, respectively. **(c) **mER^low ^MCF-7 cells treated with E_2_. **(d) **Estrogen receptor-α-negative MDA-MB-231 cells treated with E_2_. Each experiment was repeated at least three times, each time point represents 16 replicate wells, and the values are expressed as mean ± standard error. Insets: inhibition of ERK1/2 activation with U0126 upstream mitogen-activated protein kinase kinase (MEK)1/2 inhibitor. The cells were pretreated for 15 min with 40 μmol/l inhibitor (gray bars) and E_2_-induced activation of ERK1/2 was tested at activation times appropriate for each cell type (after 3, 6 and 10 min in the case of mER^high ^or 6 min in the case of mER^low ^cells). The asterisks indicate the significant reduction in ERK1/2 activation compared with cells treated only with E_2 _(black bars).

In mER^high ^MCF-7 cells, ERK1/2 activation with 1 pmol/l E_2 _was fast and transient (Fig. 7a). The maximal activation was achieved after 10 min, followed by a rapid decline in phosphorylated ERK1/2. However, continued incubation with E_2 _(1 hour) resulted in the reactivation of ERK1/2. To test whether this signal was initiated at the membrane, the impeded ligand E_2_-peroxidase was applied to the cells at steroid concentrations that approximated the levels applied as free steroid in the previous experiments (Fig. 7b). To eliminate any free steroid present, just before use we pretreated the E_2_-peroxidase with dextran-coated charcoal under conditions that remove more than 99% of free hormone [9]. The resulting maximal level of ERK1/2 activation was slightly higher than for treatment with the free ligand, but the peak time of activation was the same. Again, a recurrent later ERK activation was observed.

Cells with lower levels of mER-α (Fig. 7c) also had the capacity for fast and transient activation of ERK1/2, but this smaller activation peak appeared at 6 min after 1 pmol/l E_2 _treatment. The levels of phosphorylated ERK declined between 10 and 30 min of E_2 _treatment as they had with mER^high ^cells; however, at longer incubations (1 hour) no reactivation was seen but rather a further ERK1/2 apparent dephosphorylation was observed. This implies that higher levels of mER-α associated with more robust early ERK activation are also responsible for the sustained ERK reactivation at the later stage.

The inhibitor of the upstream MEK1/2, namely U0126, was effective in inhibiting ERK1/2 activation in both types of MCF-7 cells (Fig. 6a and 6c, insets), verifying that the values we measured in our plate assay were from MEK-phosphorylated ERK. In the MDA-MB-231 ER-α-negative cell line (Fig. 7d), E_2 _could not significantly activate ERK1/2, confirming that ER-α is necessary for ERK activation during this 60 min time period.

### Dose-dependent activation of ERK1/2 by 17β-estradiol is influenced by the level of membrane ER-α expression

**Figure 8:**
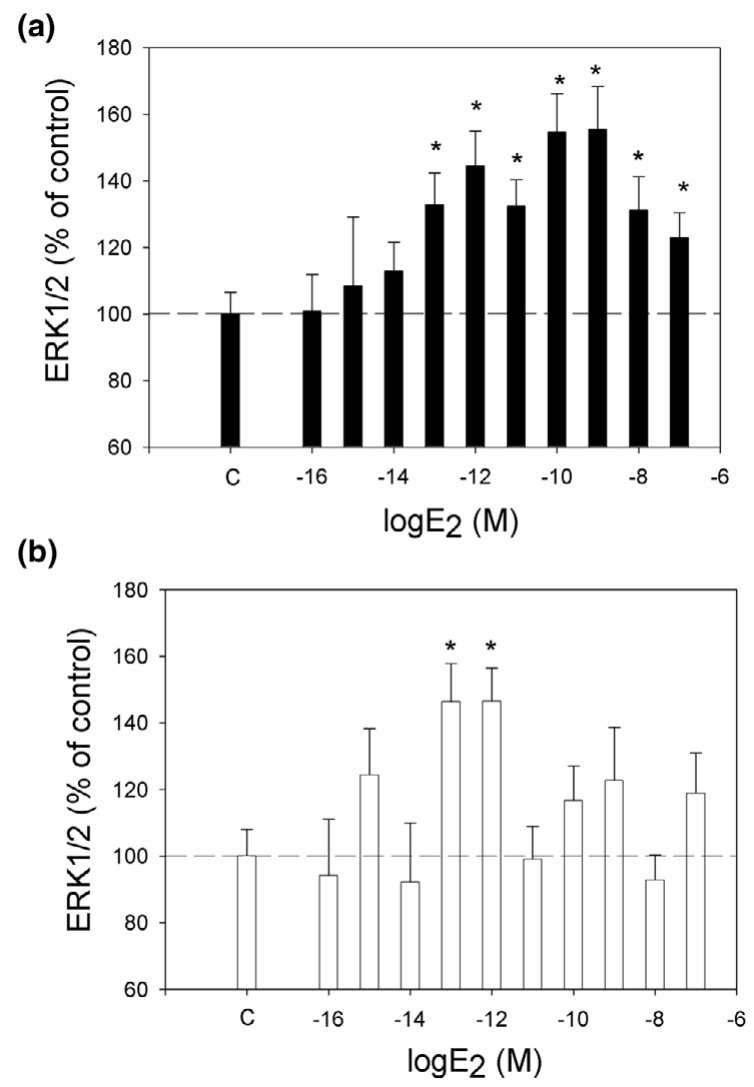
Dose-dependent 17β-estradiol (E_2_) activation of extracellular signal-regulated kinase (ERK)1/2 in membrane estrogen receptor (mER)-α-enriched (mER^high^) versus mER-α-depleted (mER^low^) MCF-7 cells. Cells were incubated with different E_2 _concentrations at peak activation times: **(a) **10 min in the case of mER^high ^and **(b) **6 min in the case of mER^low^. The experiments were repeated three times, each with 16 replicates; values are expressed as mean ± standard error. The asterisks indicate significant differences from the vehicle controls (C).

In mER^high ^cells, the ability of E_2 _to induce ERK activation was biphasic with respect to dose at the 10 min time point (early response peak). ERK phosphorylation was stimulated at a wide range of concentrations from 0.1 pmol/l to 100 nmol/l E_2_, although the highest E_2 _concentrations resulted in less phosphorylation (Fig. 8a). In mER^low ^cells a biphasic response was also seen, but the only effective concentrations were 0.1 and 1 pmol/l for the 6 min (early and only) response peak (Fig. 8b).

### Physiologic significance of early ERK1/2 activation

**Figure 9:**
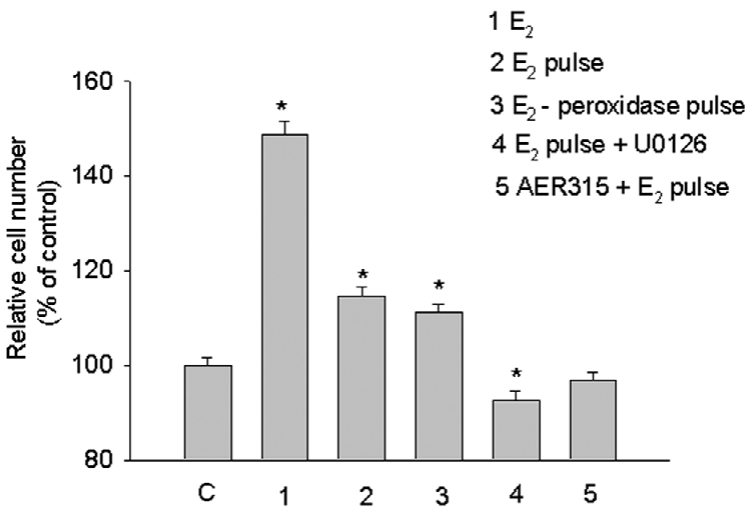
Pulsed effect of 17β-estradiol (E_2_) and conjugated E_2 _on cell proliferation. MCF-7 membrane estrogen receptor (mER)-α-enriched (mER^high^) MCF-7 cells were treated for 5 days (bar 1) or 10 min with 1 pmol/l E_2 _(bar 2) or E_2_-peroxidase containing the equivalent of 1 pmol/l E_2 _(bar 3). In bar 4, pretreatment with U0126 (40 μmol/l) for 15 min was followed by 10 min treatment with E_2_. Bar 5 shows blockage of mER-α by incubating the cells for 1 hour at room temperature with 1 μg/ml of a ligand binding domain-specific antibody and the subsequent effect of 10 min incubation with E_2_. These experiments were repeated three times, each with 40 well replicates; values are expressed as mean ± standard error. The asterisks indicate significant differences from the controls (C – ethanol vehicle, inhibitor, or antibody treatment alone).

Long-term treatment of mER^high ^MCF-7 cells with 1 pmol/l E_2 _resulted in substantial stimulation of proliferation (Fig. 9). A 10 min short pulse treatment also resulted in significant although lower stimulation of proliferation. The same level of stimulation was achieved with both E_2 _and E_2_-peroxidase presented for a short pulse. E_2_-induced proliferation was prevented with MEK inhibitor (U0126) as well as with a specific ER-α antibody recognizing the ligand binding domain. These results are consistent with the participation of mER-α and ERK1/2 in the cell proliferation response.

### Phosphatase inhibitors differentially affect ERK activation in MCF-7 cells enriched and depleted for membrane ER-α

**Figure 10:**
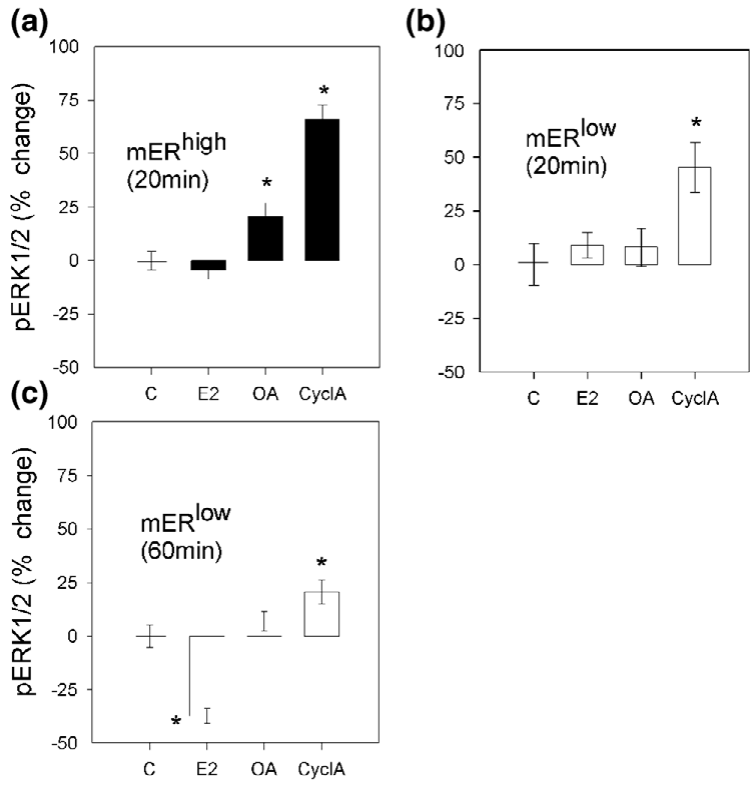
Effects of phosphatase inhibitors on extracellular signal-regulated kinase (ERK)1/2 dephosphorylation. Cells were pretreated for 1 hour with different phosphatase inhibitors: 50 nmol/l okadaic acid (OA) or 3 μmol/l cyclosporin A (CyclA). Then, ERK1/2 phosphorylation was determined after 1 pmol/l E_2 _stimulation for **(a, b) **20 min or **(c) **60 min. Black bars are membrane estrogen receptor (mER)-α-enriched (mER^high^) MCF-7 cells; open bars are mER-α-depleted (mER^low^) MCF-7 cells. The data are averages of two or three experiments, each with 24 replicates; values are expressed as mean ± standard error. The asterisks indicate significant differences from the vehicle control-treated samples with corresponding inhibitor included.

We next asked whether the observed decrease in phosphorylated ERK1/2 after 20 min in both subpopulations of cells, and the continued low phosphorylation levels after 60 min in mER^low ^cells, could successfully be abrogated with specific phosphatase inhibitors (Fig. 10). We tested inhibitors of protein phosphatase (PP)1, PP1A, and PP2B. These phosphatases can be considered principal enzymes of this class, based on their general abundance and broad specificity [30]. Okadaic acid (OA), an inhibitor of PP1 and PP1A, and cyclosporin A, an inhibitor of PP2B, were both able to reverse the ERK inactivation in mER^high ^cells (Fig. 10a). In mER^low ^cells, both the 20 min and continued 60 min dephosphorylation were abrogated only with the PP2B inhibitor (Fig. 10b and 10c). Because of the known apoptotic effect of OA at some concentrations [31], it is important to stress that we applied it at a low concentration (50 nmol/l). In addition, OA does not have toxic effects in short-term incubations [32]. These results suggest that dephosphorylation of ERKs is an important component of their process of action and that coordinated phosphorylation/dephosphorylation is required for strong signaling through this pathway.

### Rapid effects of ICI182,780 and 17α-estradiol on ERK1/2 activation

**Figure 11:**
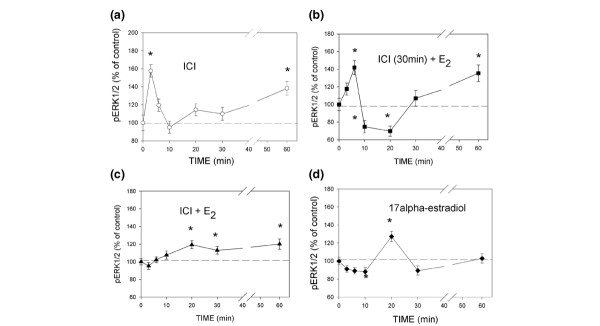
Effects of ICI182,780 (ICI), ICI + 17β-estradiol (E_2_), and 17α-estradiol on extracellular signal-regulated kinase (ERK)1/2 activation in membrane estrogen receptor-α-enriched (mER^high^) MCF-7 cells. **(a) **Time course of ERK1/2 activation by 1 μmol/l ICI. **(b) **Pretreatment with ICI (1 μmol/l) for 30 min followed by 1 pmol/l E_2 _stimulation in the continued presence of ICI. **(c) **ICI (1 μmol/l) and E_2 _(1 pmol/l) applied simultaneously. **(d) **Time course of 17α-estradiol (10 nmol/l) activation of ERK1/2. All experiments were repeated at least three times with 24 well replicates/experiment; the averaged values ± standard error are presented. The asterisks indicate significant differences from vehicle controls.

To characterize the pharmacologic properties of ERK activation, we used mER^high ^cells to study the effectiveness of the potent antiestrogen ICI182,780 (1 μmol/l) and the inactive E_2 _stereoisomer 17α-estradiol (10 nmol/l). We used concentrations of these compounds shown by others to be effective in inhibiting the transcriptional activity of E_2_. We had also previously shown that a 10 nmol/l 17α-estradiol concentration could elicit another type of nongenomic estrogenic effect in our GH_3_/B6/F10 pituitary tumor cell model – rapid prolactin release [15]. ICI182,780 alone induced an activation pattern very similar to that seen with E_2 _but with an earlier first peak (Fig. 11a). A 30 min ICI182,780 preincubation before a subsequent 1 pmol/l E_2 _challenge did not significantly change the E_2 _activation pattern, although the first peak again appeared at 6 min (the ICI182,780 pattern) and there was a much more pronounced inactivation at 10 and 20 min (compare Fig. 11b with Figs 7a and 11a). However, simultaneous application of ICI182,780 (1 μmol/l) and E_2 _(1 pmol/l) blunted the response and altered the kinetics of ERK phosphorylation, shifting the now single weak activation to later times (20–60 min; Fig. 11c). The E_2 _stereoisomer (17α-estradiol) provoked a slightly delayed and blunted response also, but with some other interesting features (Fig. 11d). A significant dephosphorylation occurred before the major activation peak, a return to baseline phosphorylation levels followed the 20 min activation peak, and no second activation peak occurred at 60 min.

## Discussion

In the late 1970s, Pietras and Szego [33] reported the presence of high-affinity binding sites for E_2 _associated with the plasma membranes of the MCF-7 human breast cancer cell line. Since that time few laboratories have followed up on this finding [34,35] until very recently, when more detailed studies started to emerge. However, none of these studies established a correlation between the level of identified mER-α expression and its functions.

To address this issue we used immunopanning followed by FACS to separate MCF-7 cells into two subpopulations that were enriched and depleted for mER-α expression. We then used several approaches to assess the appearance and levels of mER-α in these two subpopulations. These studies demonstrated that MCF-7 cells are heterogeneous with respect to mER-α expression, and that difficulties in reproducibly demonstrating nongenomic estrogenic effects in these cells could in part be due to the dilution of responding cells in the largely nonresponsive total cell population. We were able to obtain two distinct cell subpopulations without applying plasmid-based transfection manipulations to over-express receptor (with probable accompanying altered regulatory parameters). Our experimental model avoids changes in stoichiometry of the multiple interacting proteins that are involved in steroid actions. Because our mER^high ^and mER^low ^cells were not clonally derived (from a single cell), the observed differences between these subpopulations cannot be attributed to clonal variations.

A characteristic punctate staining for mER (earlier reported for GH_3_/B6/F10 cells [22]) was also detected on the surface of most of our mER^high ^MCF-7 cells. MCF-7 mER^low ^cells also had occasional punctuate staining, but at a much lower level. Because it is difficult and time-consuming to quantify this staining via immunocytochemistry, we modified our previously developed 96-well plate immunoassay [23] to measure both membrane and intracellular receptors in breast cancer cells, and to quantitate the relative amounts in these two receptor subpopulations. This assay must be tailored to specific cell types to ensure preservation of the membranes (which have quite different compositions in different cell types) and to optimize for different antibody labeling systems. Although mER-α levels differed between the two cell types named for these differences (high versus low), we discovered that the two subpopulations had the same quantity of total receptor (measured in permeabilized cells). This finding is consistent with intracellular and membrane fractions of ER-α being from the same ER pool, but with a different balance of subcellular distribution.

There is disagreement in the literature on the expression of caveolin-1 and -2 in MCF-7 cells. To test whether mER is localized in caveolar membranes, we had first to resolve this uncertainty. Some have reported that in MCF-7 cells caveolin-1 is downregulated and only caveolin-2 is expressed [36,37]. Others reported that caveolin-1 could be upregulated and downregulated in MCF-7 cells in concert with the cells' ability to develop drug resistance [38]. Some studies used transient transfection to re-express the missing caveolin-1 and test for its function in signaling [7,39]. Apparently, there are several different subpopulations of MCF-7 cells growing in different laboratories [40,41]. Our MCF-7 cells expressed both caveolin-1 and -2, and ER-α was associated with the same gradient fractions in which most of the caveolin proteins were detected. A 67 kDa ER and a variant of lower molecular weight (52 kDa) were detected in both the cell lysate and the caveolar membrane gradient fractions. Until recently the lower molecular weight ER variants were thought to be proteolytic fragments, but evidence that some of those molecules are truncated products of the full-length ER-α was recently presented. It is well documented that in MCF-7 breast cancer cells ER-α mRNA can undergo alternative splicing, generating transcripts with single, double, or multiple exon deletions [42], and that a 52 kDa protein is translated from the predominant splice variant mRNA that is missing exon 7 [43]. Others have identified a 46 kDa variant in human endothelial cells [44] and in MCF-7 cells [45,46]. Some evidence suggests that the 46 kDa receptor is the major functional membrane form of ER [44]; however, the precise functions of the truncated ER-αs are still under investigation.

The traditional detection and quantitation of phosphorylated MAPKs via Western blot analysis is laborious and somewhat arbitrary in the designation of appropriate densitometric backgrounds for comparison, especially in situations where the significant activation response is not large. We developed an enzyme-linked immunosorbent assay to deal with these experimental problems using fixed cells [47], which allowed convenient testing of large numbers of different conditions. Ours is the first report that different levels of mER in MCF-7 cells influence the different temporal and dose-dependent estrogen-induced phosphorylations of ERKs. The subpopulation of cells with high mER levels exhibited early and more robust activation, peaking at 10 min with a reactivation at 60 min, whereas the subpopulation of cells with low mER levels were only capable of weakly activating ERKs at one early time point (6 min). Furthermore, in the ER-α-negative cell line MDA-MB-231, E_2 _could not activate these kinases. The physiologic significance of early ERK1/2 activation was confirmed by showing that short pulse E_2 _treatment also stimulated cell proliferation. We and others [46] have confirmed that mER-α in breast cancer cells is responsible for this effect by showing that E_2_-peroxidase can stimulate proliferation and that this effect can be abolished by prior blocking of ER-α with ligand-binding domain-specific antibody.

The inability to activate ERKs with E_2 _in an ER-α-negative cell line, and the ability to do so in MCF-7 ER-α-positive cells, was assigned by others to the orphan G-protein-coupled receptor GPR30 [48]. However, recent studies with antisense GPR30 knockdowns revealed that E_2_-stimulated MCF-7 cells proliferate as well as cells with normal levels of GPR30 [49,50]. ERK activation associated with the ability of these cells to respond to estrogens by proliferating thus does not appear to be a function of GPR30 levels.

Different levels of mER also determined the E_2 _dose-dependent phosphorylation of ERKs. Cells with low levels of mER responded to a limited range of concentrations (10^-13 ^to 10^-12 ^mol/l). However, mER^high ^cells responded to a much wider range of E_2 _concentrations (10^-13 ^to 10^-7 ^mol/l) with a declining (but still significant) ERK activation at 10–100 nmol/l E_2_. This finding corresponds to our observation that 10 nmol/l and higher E_2 _decreases cell proliferation in mER^high ^MCF-7 cells, and is consistent with the idea that E_2 _induces cAMP-activated protein kinase A inhibition of MAPK pathways at these higher concentrations (see accompanying paper [28]). Activation of ERKs has been linked to the proliferative cellular response [51]. Our accompanying report [28] addresses other rapid estrogen-elicited signaling responses that, in concert with ERK activation, can affect and perhaps balance cell proliferation responses.

The classical antiestrogen ICI182,780 has been well defined as an antagonist of the action of estrogen at the transcriptional level. In our studies this compound was also a potent and rapid ERK activator. With different kinetics, the transcriptionally inactive 17α-estradiol also activated ERKs. It has been demonstrated that pure antiestrogen–ER complex can bind to estrogen response elements, but that the resultant transcriptional unit is inactive [52]. This binding and inactivation is generally used for pharmacological identification of ER-α participation in gene transcription. However, in a yeast reporter system antiestrogens (ICI182,780 and tamoxifen) induce ER dimerization and transcriptional activity [53]. Thus, although the published data disagree, it remains possible that antiestrogen–ER complexes can exert rapid effects such as induction of ERK phosphorylation and other signaling effects [54,55].

Other differences between findings may stem from effects assessed at different time points [56-58]. The length of time between estrogen or antiestrogen administration and kinase assessment can clearly influence outcomes, as evidenced by our time- and compound sequencing-dependent changes in ERK activation. It is also known that lengthy exposures to ICI182,780 can dramatically decrease ER-α protein levels [47,52], which could explain the decreased ERK activation observed in some studies. Long-term estrogen-deprived MCF-7 cells express higher levels of ER than do wild-type MCF-7 cells and are hypersensitive to E_2_; ICI182,780 did not alter the pattern of response to E_2_-stimulated growth in these cells [59,60]. To reconcile this observation with the expected inhibitory effect of ICI182,780, the authors suggested that ICI182,780 blocked only the effect of the residual E_2 _coming from plastic tissue culture flasks, without affecting the added E_2_. We can speculate that the observed effect of ICI182,780 was genuine and resulted from potentially increased levels of mER in long-term estrogen deprived MCF-7 cells, because our previous studies suggested that serum deprivation elevates mER-α levels [61]. In other cell types ICI182,780 behaved as a potent agonist of ERK1/2 activation (e.g. in rat cerebellar neurons expressing plasmid-generated ERs [60]), or as a partial ER agonist (in the superficial stroma and glandular epithelium of the sheep uterine endometrium [62]). It is likely that subtle differences in the shape of estrogen- and antiestrogen-liganded ERs, whether nuclear or membrane, present different interaction platforms for a variety of co-modulators, and that each signaling pathway must be considered in the context of a particular cell type's repertoire of partnering proteins.

In addition to having positive effects by itself, ICI182,780 was able to alter the E_2 _response differently, dependent on the timing of its administration. If the cells were pretreated for only 30 min (the time point when ICI182,780 alone did not change basal ERK activation), then the initial E_2 _activation and the late re-activation at 60 min were preserved, whereas the after-peak deactivation was enhanced. If ICI182,780 and E_2 _were added simultaneously, then the activation was delayed to 20 min, but weakly persisted up to 1 hour. Similar observations that ICI182,780 can change the time of ERK activation were reported in the case of a human thyroid carcinoma cell line, in which it reduced the 30 min to 1 hour activation, but enhanced later sustained activation at 6 hours [63]. The same authors showed that, in differentiated thyroid gland carcinoma cells (XTC133), addition of ICI182,780 induces a small decrease in the sustained ERK phosphorylation. Thus, in addition to demonstrating that ICI182,780 is not just an antagonist for this response, these studies and ours may point to cell-specific differences in ERK regulation.

Phosphorylations leading to activation and subsequent inactivation of proteins are important regulatory mechanisms for control of cell growth and differentiation [64,65]. Although ERKs are thought to play a key role in cell proliferation, it has been suggested that persistent activation of ERKs might result in cell cycle arrest and differentiation in PC12 cells, or cell growth inhibition and apoptosis in normal rat hepatocytes and several human tumor cell lines [65]. However, a detailed time course and transient ERK activations, interspersed with phosphatase-mediated inactivations, are rarely addressed. We (in the present study) and others [66] showed that the serine/threonine phosphatases PP1 and PP2A participate in the dephosphorylation of ERKs. It is interesting that OA (a PP1 and PP2A inhibitor) was more effective in mER^high ^cells, whereas cyclosporin A (a PP2B inhibitor) was almost equally efficient in both cell populations. These data suggest that the level of mER-α expression/activation can be associated with activation of different types of ERK-modulating phosphatases. Other phosphatases, such as tyrosine phosphatase and dual specificity phosphatases (which dephosphorylate tyrosine, threonine, and serine residues), have been implicated in ERK dephosphorylation [67,68] and will be the subject of our future studies.

Additional studies of ERK activations, deactivations, and stability will be needed before we can formulate a more global picture of the post-translational modifications that lead to function of this important group of regulators in proliferation and differentiation. However, it is clear that nongenomic estrogen actions and the membrane receptors through which they act participate in this regulation.

## Conclusion

E_2_-induced changes in breast cancer cell number [28] can be directly related to ERK1/2 activation/deactivation patterns and interacting signaling mechanisms. The differential behavior of cell lines expressing different levels of mER-α suggests a role for this receptor in the temporal coordination of phosphorylation/dephosphorylation events affecting the mitogen-activated kinases ERK1 and ERK2.

## Abbreviations

CV = crystal violet; DCSS = medium with 4 × dextran-coated charcoal-stripped serum; E_2 _= 17β-estradiol; EGF = epidermal growth factor; ER = estrogen receptor; ERK = extracellular signal-regulated kinase; FACS = fluorescence-activated cell sorting; OA = okadaic acid; MAPK = mitogen-activated protein kinase; MEK = mitogen-activated protein kinase kinase; mER = membrane estrogen receptor; pNp = paranitrophenol; PP = protein phosphatase; tER = total estrogen receptor.

## Competing interests

The author(s) declare that they have no competing interests.
